# Serum anti-malondialdehyde-acetaldehyde IgA antibody concentration improves prediction of coronary atherosclerosis beyond traditional risk factors in patients with rheumatoid arthritis

**DOI:** 10.1038/s41598-022-14954-9

**Published:** 2022-06-22

**Authors:** Hannah E. Lomzenski, Geoffrey M. Thiele, Michael J. Duryee, Sheau-Chiann Chen, Fei Ye, Daniel R. Anderson, Ted R. Mikuls, Michelle J. Ormseth

**Affiliations:** 1 Vanderbilt University Medical Center, Nashville, TN USA; 2 University of Nebraska Medical Center, Omaha, NE USA; 3 VA Nebraska-Western Iowa Health Care System, Omaha, NE USA; 4 Tennessee Valley Healthcare System, U.S. Department of Veterans Affairs, Nashville, TN USA

**Keywords:** Rheumatoid arthritis, Risk factors, Cardiology, Biomarkers

## Abstract

Patients with rheumatoid arthritis (RA) have increased atherosclerosis; oxidative stress may be a contributor. Oxidative stress produces immunogenic malondialdehyde-acetaldehyde (MAA) protein adducts and anti-MAA antibodies are detectable in human serum. We hypothesized that anti-MAA antibody concentrations are associated with coronary atherosclerosis in RA patients. Serum concentrations of anti-MAA antibodies (IgA, IgG, and IgM) were measured in 166 RA patients using ELISA cross-sectionally. Relationship between anti-MAA antibody concentrations and cardiovascular and metabolic measures and predictive accuracy of anti-MAA antibodies for presence of coronary artery calcium (CAC) and high CAC (≥ 300 Agatston units or ≥ 75^th^ percentile) were assessed. Only serum IgA anti-MAA antibody concentration was associated with increased CAC, insulin resistance, and decreased high-density lipoprotein particle number. When added as an interaction term with ACC/AHA 10-year risk score plus high-sensitivity C-reactive protein, IgA anti-MAA antibody concentration improved the C-statistic for prediction of any CAC and high CAC compared to ACC/AHA 10-year risk score plus hs-CRP alone. IgA anti-MAA concentration is associated with multiple cardiovascular risk factors and modifies the relationship between ACC/AHA 10-year risk score and CAC in RA patients. IgA anti-MAA concentration could assist in prediction of atherosclerotic CVD and risk stratification when added to standard measures of cardiovascular risk.

## Introduction

Rheumatoid arthritis (RA) is associated with increased oxidative stress^1,2^. When tissues are exposed to higher levels of oxidative stress, there is increased lipid peroxidation, which results in the formation of malondialdehyde (MDA)^3^. MDA degrades into acetaldehyde (AA); together, MDA and AA then react with proteins to form the more stable protein adduct malondialdehyde-acetaldehyde or MAA^3^. MAA-adducted proteins are highly immunogenic and are found in the serum of patients with various systemic inflammatory diseases, including RA^4–7^. Antibodies against these MAA adducts, known as anti-MAA antibodies, can be detected in human serum^8^, and increase significantly 2–3 years prior to RA diagnosis^9^. Furthermore, MAA-adducted proteins are also present in the vascular lesions of atherosclerosis^10^ and may be a link between RA and accelerated atherosclerosis.

In patients with established RA, the prevalence and severity of coronary atherosclerosis is increased compared to age, race, and sex-matched control subjects^11^. While some studies indicate decreasing incidence of cardiovascular events in patients with RA in comparison to the general population^12^, RA patients experience poorer long-term outcomes after myocardial infarction, with 58% higher mortality rate by 5 years^13^. Unfortunately, traditional cardiovascular risk algorithms routinely underestimate cardiovascular disease (CVD) risk in patients with RA^14–16^. Even RA-specific risk scores, which incorporate disease activity and inflammatory markers, are unable to accurately predict cardiovascular risk, further underscoring the need for more accurate predictive tools in patients with RA^17^.

Elevated concentrations of anti-MAA antibodies in the serum are significantly associated with coronary artery disease in the general population^18^, raising the possibility of their use as predictive biomarkers for atherosclerotic disease in RA. Additionally, significantly higher concentrations of anti-MAA antibodies are demonstrated in both the serum and synovium of patients with RA when compared to controls^6^. Thus, we hypothesized that in patients with RA increased serum concentrations of anti-MAA antibodies would be associated with coronary artery atherosclerosis.

## Methods

### Study population

We conducted a cross-sectional study of 166 patients with RA who were previously characterized for CV risk^1,11^. Recruitment and study procedures have been detailed previously^11^. All subjects were greater than 18 years of age and met the 1987 revised classification criteria for RA. Race and ethnicity were self-reported by participants. The study was approved by the Vanderbilt University Medical Center Institutional Review Board. All subjects provided written informed consent and all research was performed in accordance with relevant guidelines and regulations.

### Clinical measurements and scores

Body mass index (BMI) was calculated as weight in kilograms divided by height in meters^2. Erythrocyte sedimentation rate (ESR), high-sensitivity C-reactive protein (hs-CRP), and fasting serum total cholesterol, low-density lipoprotein (LDL) cholesterol, high-density lipoprotein (HDL) cholesterol, triglycerides, glucose, and insulin concentrations were measured at the Vanderbilt University Medical Center Laboratory. HDL particle number was measured by nuclear magnetic resonance (Liposcience, Inc, Raleigh, NC). The homeostatic model assessment (HOMA) for insulin resistance was calculated as fasting plasma glucose (mmol/l) * fasting serum insulin (mU/l)/22.5^19^.

The 28-joint disease activity score using ESR (DAS28-ESR) was used to assess RA disease activity^20^. The American College of Cardiology/American Heart Association (ACC/AHA) 10-year risk score was calculated on each patient, using their previously defined measurements of age, race, sex, total cholesterol, HDL cholesterol, systolic blood pressure, and presence of diabetes, current tobacco use, and/or anti-hypertensive use^21^.

Coronary artery calcium (CAC) scores were calculated using electron beam computed tomography imaging and quantified in Agatston units as previously described^11^. High CAC was defined as a score ≥ 300 Agatston units or ≥ 75^th^ percentile for age, sex, and ethnicity^22^. Low CAC was defined as not meeting criteria for high CAC, thus also including those with no CAC.

Serum samples were used to measure immunoglobulin (Ig)-M, IgG, and IgA antibodies to MAA by enzyme-linked immunosorbent assay, as previously described in detail^7,10^. In brief, MAA adducted human albumin (MAA-Alb) or albumin (Alb) was used to coat ELISA plates. Plates were first incubated at 4 °C overnight, washed, blocked with 2% bovine serum albumin, and then re-incubated with patient serum at a 1:1000 dilution at 37 °C for 1 h. The plates were treated with a secondary goat anti-human antibody specific for IgM, IgG, or IgA. Plates were developed using tetramethylbenzine substrate, with absorbance determined at 450 nm. The serum concentrations of the IgM, IgG, and IgA anti-MAA antibodies are presented as arbitrary units (AU) relative to a standard curve. Serum anti-Alb concentration was subtracted from anti-MAA-Alb concentration to represent anti-MAA antibody concentration.

### Biostatistics

Descriptive statistics are presented as median [interquartile range (IQR)] for continuous variables and number (percent) for categorical variables. Wilcoxon’s rank sum tests were used to compare continuous variables and Pearson’s chi-square test to compare categorical variables. Spearman correlation was used to assess correlations between continuous variables. Anti-MAA IgA was natural-log (ln-) transformed when entered into the models. To classify presence versus absence of CAC and presence of high versus low CAC, binary logistic regression was used. In the models presence versus absence of CAC and presence of high versus low CAC were outcomes of interest. Predictors were ACC/AHA 10 years risk score, ln-transformed anti-MAA IgA, and hsCRP, and separately ACC/AHA 10 years risk score and ln-transformed anti-MAA IgA as an interaction and hsCRP. To examine the accuracy of the models, the C-statistic was calculated (equivalent to the area under the receiver operating characteristic curve) with bootstrapping for internal validation of each fitted logistic regression model. Nested models were compared using the likelihood ratio test. All analyses were performed using R version 3.6.1 or IBM SPSS Statistics version 26.

## Results

### Clinical characteristics

The median [interquartile range (IQR)] age of the 166 RA patients was 54 years [45, 63 years]. Approximately 69% were female and 89% were Caucasian (Table 1). Most patients (72%) were seropositive for rheumatoid factor. Disease activity was mostly low to moderate with median DAS28-ESR score of 3.88 units [2.64, 4.84 units]. A majority of patients were taking methotrexate (72%) and corticosteroids (54%) while 20% were taking an anti-TNFα agent. Demographics based on presence or absence of high CAC is presented in the Supplemental Table 1.

**Table 1:** Demographics and clinical features.

	All RA (*N* = 166)	Without CAC (*N* = 79)	With CAC (*N* = 82)	*P* value
Age, years	54 [45, 63]	48 [40, 54]	60 [54, 69]	< 0.001
Sex, # female	114 (69)	64 (81)	47 (57)	0.001
Race, # Caucasian	150 (88)	68 (86)	75 (91)	0.28
DAS28-ESR, score	3.88 [2.64,3.4.84]	3.46 [2.39, 4.40]	4.05 [2.84, 5.01]	0.04
Rheumatoid factor, # positive	114 (72)	52 (69)	58 (72)	0.66
hs-CRP, mg/dl	4.0 [1.2, 11]	3.00 [1.00, 9.50]	5.00 [2.00, 12.50]	0.09
Waist/hip ratio, units	0.88 [0.81, 0.95]	0.84 [0.78, 0.92]	0.91 [0.83, 0.99]	< 0.001
Body Mass Index, kg/m2	28.3 [24.0, 33.1]	28.30 [24.02, 33.51]	28.34 [23.71, 31.53]	0.34
Systolic BP, mmHg	133 [118, 146]	129 [116, 139]	137 [121, 151]	0.004
Diastolic BP, mmHg	75 [67, 82]	73 [67, 80]	76 [69, 85]	0.13
Current smoker, # yes	40 (24)	17 (22)	22 (27)	0.44
Total cholesterol, mg/dL	184 [156, 210]	183 [157, 204]	188 [154, 214]	0.70
LDL-cholesterol, mg/dL	111 [88, 134]	105 [89, 134]	115 [90, 136]	0.44
HDL-cholesterol, mg/dL	43 [37, 54]	44 [38, 55]	43 [36, 53]	0.32
Triglycerides, mg/dL	111 [80, 158]	108 [81, 149]	112 [80, 164]	0.58
CAC score, Agatston units	2.70 [0.00, 150.30]	0.00 [0.00, 0.00]	148.80 [40.80, 470.30]	< 0.001
Statins, # user	21 (12)	6 (7)	15 (18)	0.04
NSAIDs, # user	54 (33)	29 (37)	25 (30)	0.40
Corticosteroid, # user	89 (54)	42 (53)	45 (55)	0.83
Methotrexate, # user	118 (72)	61 (77)	54 (66)	0.11
Hydroxychloroquine, # user	42 (25)	23 (29)	17 (21)	0.22
Leflunomide, # user	29 (18)	13 (16)	16 (20)	0.61
Anti-TNFα, # user	33 (20)	17 (22)	14 (17)	0.47

### Anti-MAA antibody concentrations based on presence of any or high CAC

**Figure 1:**
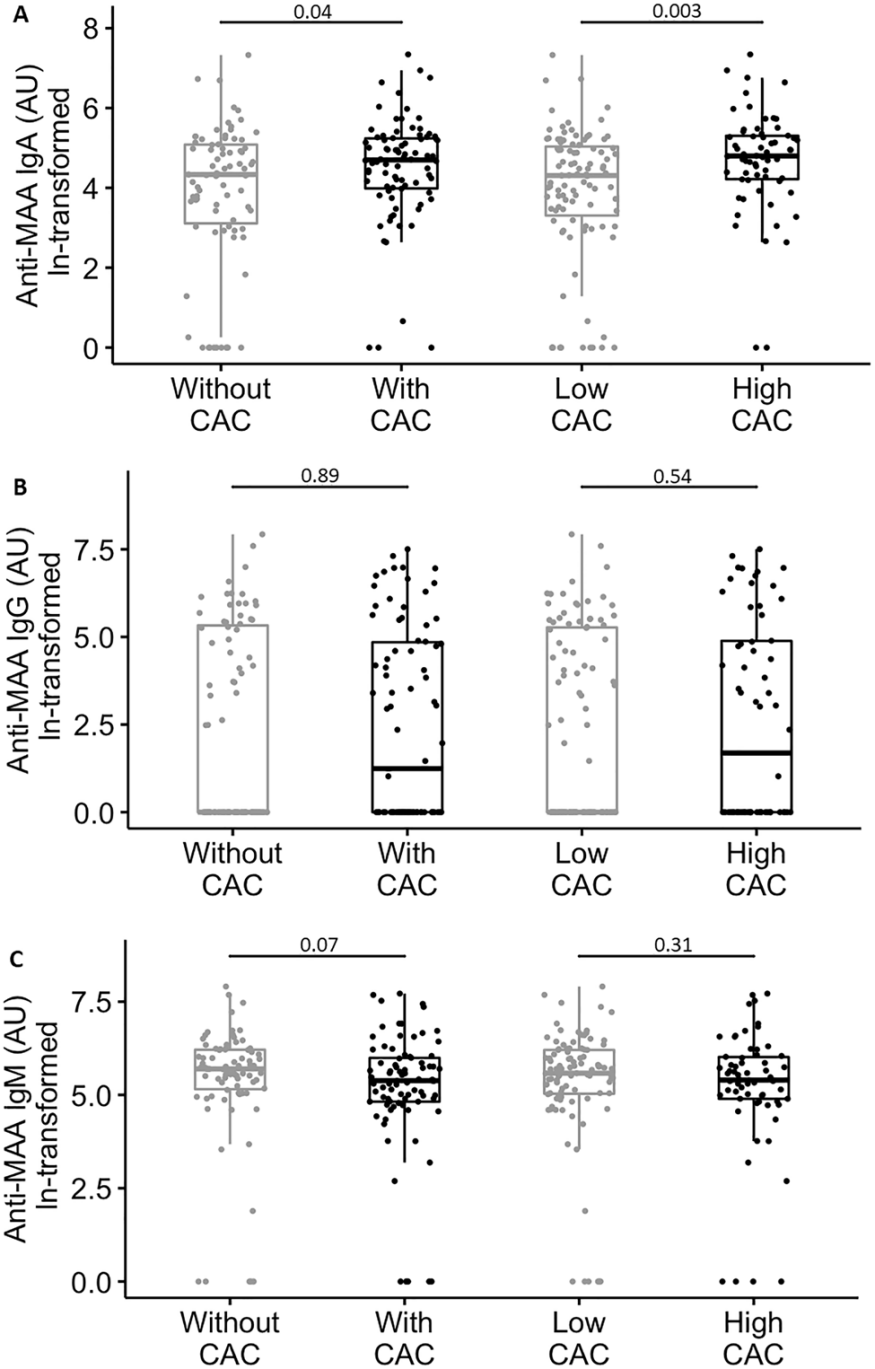
Plots of anti-MAA antibody isotype concentrations based on coronary artery calcium in patients with rheumatoid arthritis. Box demonstrates the median, upper and lower quartiles. Whiskers represent 95% confidence intervals. CAC = coronary artery calcium. High CAC = high coronary artery calcium score based on ≥ 300 Agatston units or ≥ 75th percentile for age, sex, and ethnicity. AU = arbitrary units. Serum anti-MAA IgA concentrations were significantly higher among those with CAC compared to those without CAC (Panel A, *P* = 0.04), and significantly higher among those with high CAC compared to low CAC (Panel A, *P* = 0.003). Anti-MAA IgG and IgM serum concentrations were not significantly altered based on CAC (Panels B and C, all *P* > 0.05).

Serum IgA anti-MAA concentration was significantly increased among those with detectable CAC (109 AU [53, 188 AU]) compared to those without CAC (75 AU [22, 160 AU]), *P* = 0.04. Similarly, IgA anti-MAA concentration was increased among those with high CAC (120 AU [67, 200 AU]) versus low CAC (73 AU [27, 153 AU]), *P* = 0.003. Serum IgG and IgM concentrations were not significantly altered based on presence of any CAC or high CAC (all *P* > 0.05) (Fig. 1).

### Relationship between anti-MAA antibody isotype and CVD risk factors

Higher serum IgA anti-MAA concentration was modestly associated with increased CAC (Rho 0.18, *P* = 0.02), increased insulin resistance as defined by HOMA (Rho 0.18, *P* = 0.03), and lower HDL-particle number (Rho − 0.20, *P* = 0.01) (Table 2). Higher serum IgA anti-MAA concentration was not significantly associated with BMI, waist/hip ratio, blood pressure, total cholesterol, LDL, triglycerides, hs-CRP, or DAS28-ESR (*P* > 0.05).

**Table 2:** Relationship between serum anti-MAA antibody isotype concentration and cardiometabolic measures in patients with RA.

	Anti-MAA IgA		Anti-MAA IgG		Anti-MAA IgM	
	Rho	P	Rho	P	Rho	P
Body mass index	− 0.01	0.88	− 0.01	0.95	− **0.17**	**0.03**
Waist/hip ratio	0.06	0.44	− 0.15	0.06	− **0.18**	**0.02**
Systolic BP	− 0.08	0.32	− 0.01	0.89	− 0.05	0.52
Diastolic BP	0.02	0.82	− 0.05	0.5	0.05	0.5
Total cholesterol	0.004	0.96	− 0.02	0.82	0.03	0.67
LDL-cholesterol	0.11	0.18	− 0.04	0.66	0.003	0.97
HDL-cholesterol	− 0.08	0.33	0.05	0.53	**0.18**	**0.02**
Triglycerides	− **0.20**	**0.01**	− 0.04	0.65	− **0.18**	**0.02**
HDL-particle number	− **0.20**	**0.01**	− 0.95	0.22	0.02	0.83
HOMA	**0.18**	**0.03**	0.05	0.54	− 0.1	0.19
hs-CRP	0.06	0.46	0.04	0.63	− 0.13	0.09
DAS28-ESR	0.06	0.45	0.07	0.41	− 0.04	0.66
CAC score	**0.18**	**0.02**	− 0.003	0.97	− 0.09	0.26

Significant values are in [bold].

Higher serum IgG anti-MAA concentration was not significantly associated with any measured CVD risk factor, including CAC or DAS28-ESR (*P* > 0.05) (Table 2).

Higher serum IgM anti-MAA concentration was modestly associated with decreased BMI (Rho − 0.17, *P* = 0.03), decreased waist/hip ratio (Rho − 0.18, *P* = 0.02), increased HDL-cholesterol (Rho 0.18, P = 0.02), and decreased triglycerides (Rho − 0.18, *P* = 0.02) (Table 2). However, serum IgM anti-MAA concentration was not significantly associated with CAC or the remainder of measured CVD risk factors or DAS28-ESR (all *P* > 0.05).

Prediction of coronary atherosclerosis determined by presence of coronary artery calcium.

**Figure 2:**
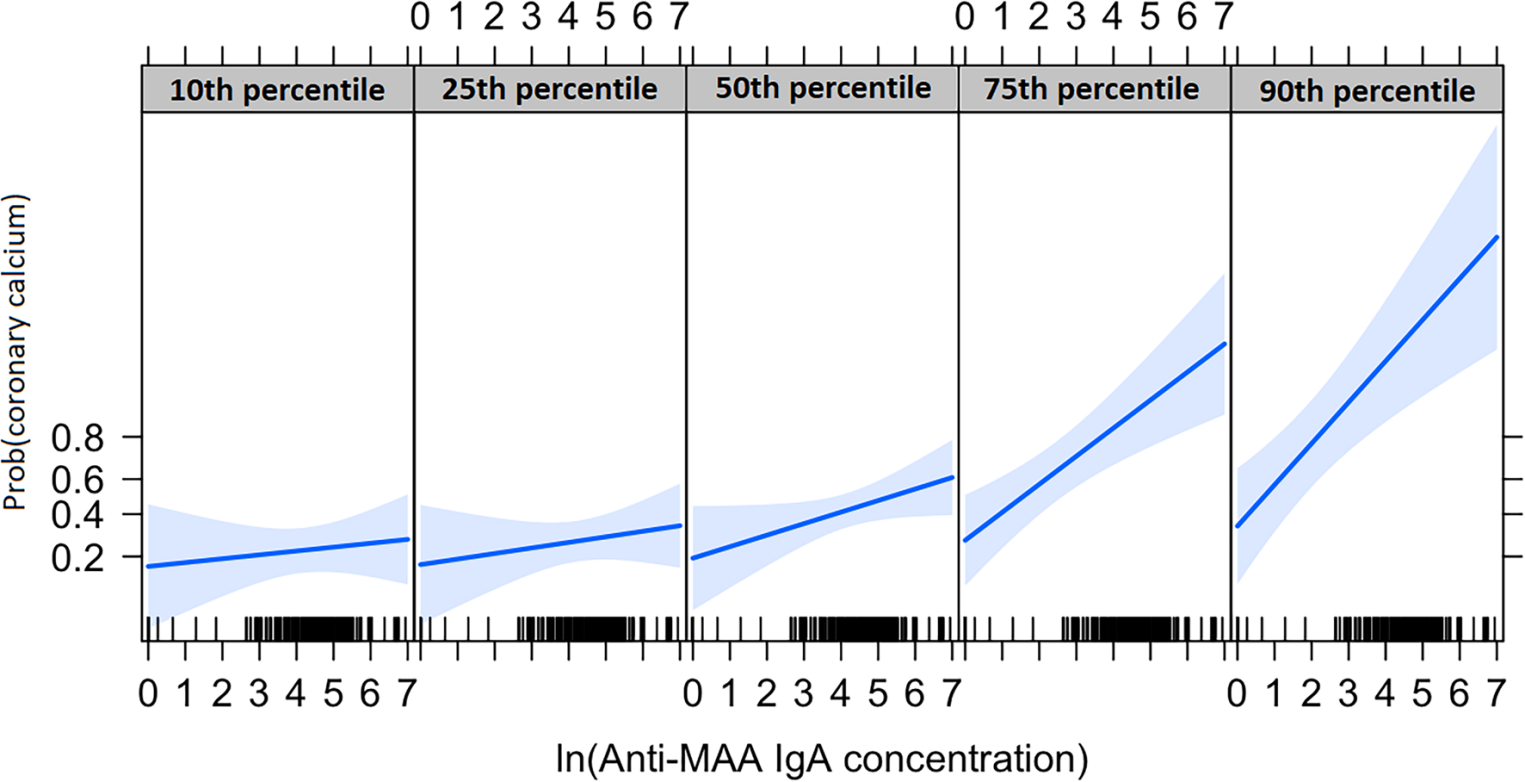
The interaction between anti-MAA IgA concentration and ACC/AHA 10-year risk score in predicting the presence of coronary artery calcium. Percentiles refer to the ACC/AHA 10-year risk score percentile of the RA patients. At higher ACC/AHA 10-year risk score, shown as 75th and 90th percentiles, presence of elevated anti-MAA IgA was associated with an amplified probability of coronary calcium.

As described above, only serum IgA anti-MAA concentration was associated with presence of CAC (Fig. 1). Anti-MAA IgA was associated with the presence of CAC independent of ACC/AHA 10-year risk score and hs-CRP (OR = 1.51 [95% CI: 1.16, 1.97], *P* = 0.002). In other words, for every one-unit increase in ln-transformed anti-MAA IgA concentration, odds of having coronary artery calcium increased 51% independent of ACC/AHA 10-year risk score and hs-CRP. Moreover, anti-MAA IgA modified the association between the ACC/AHA 10-year risk score and presence of CAC (Fig. 2, *P* = 0.004 for interaction term), indicating that in the setting of a higher ACC/AHA 10-year risk score, higher anti-MAA IgA concentration amplified the probability of having coronary calcium. Use of the interaction term improved the prediction of coronary atherosclerosis beyond ACC/AHA 10-year risk score plus hs-CRP from C-statistic = 0.835 (95% CI 0.773, 0.896) to 0.862 (95% CI 0.807, 0.917), likelihood ratio test *P* < 0.001 (Supplemental Fig. 1). However, when not used as an interaction term, anti-MAA IgA concentration did not improve the C-statistic for prediction of CAC beyond ACC/AHA 10-year risk score (C-statistic = 0.831 (95% CI 0.768, 0.893), Supplemental Fig. 1).

### Prediction of high coronary artery calcium score

Anti-MAA IgA was also associated with high CAC independent of ACC/AHA 10-year risk score and hs-CRP (OR 1.56 [95%CI 1.19, 2.02], P = 0.001). In other words, for every one unit increase in ln-transformed anti-MAA IgA concentration, the odds of having high coronary artery calcium increased 56% independent of ACC/AHA 10-year risk score and hs-CRP. In a trend similar to above with anti-MAA IgA concentration modifying the association between ACC/AHA 10-year risk score and presence of CAC, anti-MAA IgA appeared to modify the association between ACC/AHA 10-year risk score and high CAC, though not as strongly as with presence of any CAC (Supplemental Fig. 2, *P* = 0.19 for interaction term), demonstrating that in the setting of a higher ACC/AHA 10-year risk score, higher anti-MAA IgA concentration amplified the probability of having high CAC. When serum IgA anti-MAA concentration was added to the ACC/AHA 10-year risk score as an interaction term plus hs-CRP, the C-statistic for prediction of high CAC improved from 0.733 (95% CI 0.657, 0.809) to 0.761 (0.684, 0.837), likelihood ratio test *P* < 0.001 (Supplemental Fig. 3). When not used as an interaction term, anti-MAA IgA concentration still significantly improved the C-statistic for prediction of high CAC beyond ACC/AHA 10-year risk score plus hs-CRP (C-statistic = 0.746 [95% CI 0.667, 0.825], *P* < 0.001, Supplemental Fig. 3), though to a lesser extent.

To better examine the value of anti-MAA IgA for prediction of CAC compared to commonly available information to the clinician, we evaluated rheumatoid factor (RF) positivity for its ability to predict presence of CAC and high CAC among RA patients using the same models constructed above by substituting RF for anti-MAA IgA concentration. RF positivity was not significantly associated with CAC independent of ACC/AHA 10-year risk score and hs-CRP (OR 1.30 [95%CI 0.57, 2.94], *P* = 0.54), or when used as an interaction term with ACC/AHA 10-year risk score (*P* = 0.25 for interaction). RF did not significantly improve the C-statistic for prediction of CAC beyond ACC/AHA 10-year risk score plus hs-CRP whether used as predictor (C-statistic = 0.819, likelihood ratio test *P* = 0.26) or interaction term (C-statistic = 0.821, likelihood ratio test *P* = 0.16) compared to the base model (C-statistic = 0.835). Similarly, RF positivity was not significantly associated with high CAC independent of ACC/AHA 10-year risk score and hs-CRP (OR 1.03 [95%CI 1.00, 1.06], *P* = 0.07), or when used as an interaction term with ACC/AHA 10-year risk score (*P* = 0.88 for interaction). RF did not significantly improve the C-statistic for prediction of high CAC beyond ACC/AHA 10-year risk score plus hs-CRP whether used as predictor (C-statistic = 0.734, likelihood ratio test *P* = 0.50) or interaction term (C-statistic = 0.735, likelihood ratio test *P* = 0.88) compared to the base model (C-statistic = 0.733).

## Discussion

In this report, we examined the relationship between anti-MAA antibody concentrations and CAC in RA patients to ascertain their utility as a predictive biomarker. When added as an interaction term with the ACC/AHA 10-year risk score, serum IgA anti-MAA concentration improved prediction of both the presence of CAC and high CAC scores beyond using IgA anti-MAA concentration as an independent predictor. The degree of improvement in the AUC in the models is 2 to threefold greater than what was observed with using a multimarker score including CRP and other biomarkers for prediction of cardiovascular events beyond age, sex and conventional risk factors in the Framingham Heart Study^23^. We demonstrated that in the setting of a higher ACC/AHA 10-year risk score, higher anti-MAA IgA concentration was associated with augmented probability of having coronary calcium. This may also inform the biology of atherosclerosis in RA. It is possible that these MAA-adducts or the immune response generated to them act in concert with other risk factors to amplify atherosclerosis.

Atherosclerosis has long been viewed as an inflammatory disease and modification of proteins (or protein-adducts) via lipid peroxidation has been implicated in both the development of and progression of atherosclerotic disease^5,10,24,25^. In brief, the oxidative degradation of lipids by reactive oxygen species, or lipid peroxidation, yields highly reactive aldehydes such as MDA. MDA itself is unstable and can further degrade to form acetaldehyde (AA) which, in the presence of MDA, can form the unique (and far more stable) malondialdehyde-acetaldehyde adduct or MAA^26^. It is this MAA adduct that has been shown to be the immunodominant MDA-epitope, capable of inducing antibody production and enhancing T-cell proliferation, even in the absence of adjuvant^10,27^.

It is not surprising then that anti-MAA antibodies have been previously described in both the circulation of and the inflamed synovial tissues of patients with RA^7,28^, a disease state which is characterized by increased oxidative stress and lipid peroxidation^1,2^. In one study evaluating anti-MAA antibody concentrations across all isotypes (IgA, IgG, IgM) in RA patients compared to both healthy volunteers and three diseased control groups (osteoarthritis, systemic lupus erythematous, spondyloarthropathy), only the IgA isotype was found to be significantly higher^28^. Additionally, MAA adducts are elevated in RA synovial tissues compared to osteoarthritis controls with both independent localization and co-localization with mature B cells and citrullinated proteins^6,7^. These findings suggest that MAA-modified proteins may play an important role in tolerance loss and consequent immune responses in RA. However, it must be noted that the presence of MAA-modified antigen and subsequent antibody formation is not limited to RA alone. MAA adducts and anti-MAA antibodies have been implicated in other disease states associated with increased oxidative stress and lipid peroxidation including type II diabetes mellitus, smoking-related diseases (such as chronic obstructive pulmonary disease), and alcohol-related liver disease^8,29–32^.

In a study investigating the role of MAA adducts in atherosclerotic disease, Anderson et. al demonstrated a significant association between anti-MAA antibodies and both acute myocardial infarction and chronic multi-vessel obstructive CAD^18^. The IgA isotype was associated with the inflammatory response of chronic stable progressive disease whereas the IgM and IgG isotypes were associated with active progressive disease (unstable CAD or myocardial infarction). Furthermore, it was noted that the serum concentrations of both the IgM and IgG isotypes decreased 24 h post-myocardial infarction while the IgA isotype concentration remained stable, a difference thought to represent serum IgA’s inability to activate complement, form MAA-adduct immune complexes, or be cleared from circulation^18^. By demonstrating an association between increased serum concentration of the IgA, but not the IgM or IgG, anti-MAA antibody isotype and coronary artery calcium, our study’s findings in patients with RA are consistent with this previously described association between IgA and chronic, stable CAD in a non-RA cohort^18^.

Interestingly, prior studies have shown an association between the IgA isotype of rheumatoid factor (RF) with more severe, erosive disease in RA patients and increased concentrations of the IgA RF predict a poorer clinical response to TNFα inhibitors ^33–35^. A potential explanation for the central role of IgA autoantibodies in RA pathogenesis might lie in the hypothesis that autoimmunity in patients with RA originates outside of the joint, specifically within mucosal tissues such as that of the oral cavity and lung, sites where IgA-mediated immune responses are prevalent^36^. A recent study has confirmed the existence of MAA adducts (and also citrullinated proteins) within the gingival tissue of patients with periodontitis, a finding not seen in healthy controls^37^, and was increased in the lung tissue of patients with RA and interstitial lung disease (ILD) compared to those with ILD from other causes and those with emphysema^38^. When considered together, these findings support a model for autoimmunity in which environmental or microbial pathogens can trigger local mucosal inflammation and generation of neo-antigens including MAA-modified proteins. In genetically susceptible individuals this may contribute to the progression from subclinical autoimmunity to clinically apparent arthritis^9,36^.

The present study has limitations. As a cross-sectional study, only significant correlations and associations are described rather than causal relationships. Our study does not show whether serum IgA anti-MAA antibody will prove to be associated with the long-term cardiovascular events in RA patients. Future investigation into its ability to predict cardiovascular risk and the longitudinal development of cardiovascular events is needed to completely elucidate its utility in patients with RA.

In conclusion, IgA anti-MAA concentration is significantly associated with multiple cardiovascular risk factors in RA patients and modifies the relationship between ACC/AHA 10-year risk score and CAC in RA. IgA anti-MAA concentration could assist in prediction of increased atherosclerotic CVD and risk stratification when added to standard measures of cardiovascular risk.

## Supplementary Information

Supplementary Information.

## Data Availability

Deidentified data generated and used for this study are available from the corresponding author upon reasonable request, and may be requested via email, michelle.ormseth@vumc.org.
