# Supplementary material for: Serum anti-malondialdehyde-acetaldehyde IgA antibody concentration improves prediction of coronary atherosclerosis beyond traditional risk factors in patients with rheumatoid arthritis
Source: Sci Rep. 2022 Jun 22;12:10547. doi: 10.1038/s41598-022-14954-9 (PMC9217813; doi:10.1038/s41598-022-14954-9)
Supplement: Supplementary file 1 — Supplementary Information. [file 41598_2022_14954_MOESM1_ESM.docx]

**Supplemental Materials**

| **Supplemental Table 1.** Demographics and clinical features based on presence of low versus high CAC | | | |
| --- | --- | --- | --- |
|  | Low coronary calcium  N=99 | High coronary calcium  N=62 | P value |
| Age, years | 50 [43, 61] | 58 [53, 67] | <0.001 |
| Sex, # female | 75 (76) | 36 (58) | 0.02 |
| Race, # Caucasian | 89 (89) | 55 (89) | 0.97 |
| DAS28-ESR, score | 3.46 [2.39, 4.40] | 4.16 [2.92, 5.21] | 0.01 |
| Rheumatoid factor, # positive | 62 (65) | 48 (80) | 0.05 |
| hs-CRP, mg/dl | 3.00 [1.00, 8.50] | 6.50 [2.25, 16.00] | 0.006 |
| Diabetes Mellitus, type 2, # | 6 (6) | 12 (19) | 0.009 |
| HOMA, units | 1.75 [0.94, 3.00] | 3.15 [1.29, 7.49] | 0.002 |
| Waist/hip ratio, units | 0.84 [0.79, 0.92] | 0.93 [0.86, 0.99] | <0.001 |
| Body Mass Index, kg/m2 | 28.30 [23.89, 33.29] | 28.34 [23.99, 31.51] | 0.47 |
| Hypertension, # | 42 (42) | 41 (66) | 0.003 |
| Systolic BP, mmHg | 130 [116, 144] | 137 [123, 151] | 0.02 |
| Diastolic BP, mmHg | 74 [68, 81] | 76 [69, 87] | 0.34 |
| Current smoker, # yes | 20 (20) | 19 (31) | 0.13 |
| Total cholesterol, mg/dL | 183 [155, 205] | 188 [157, 217] | 0.48 |
| LDL-cholesterol, mg/dL | 107 [88, 134] | 115 [93, 136] | 0.26 |
| HDL-cholesterol, mg/dL | 43 [37, 55] | 43 [36, 51] | 0.43 |
| Triglycerides, mg/dL | 108 [80, 155] | 112 [81, 157] | 0.75 |
| CAC score, Agatston units | 0.00 [0.00, 0.00] | 345.55 [84.35, 591.68] | <0.001 |
| Statins, # user | 9 (9) | 12 (19) | 0.06 |
| NSAIDs, # user | 34 (34) | 20 (32) | 0.79 |
| Corticosteroid, # user | 55 (56) | 32 (52) | 0.63 |
| Methotrexate, # user | 77 (78) | 38 (61) | 0.02 |
| Hydroxychloroquine, # user | 26 (26) | 14 (23) | 0.60 |
| Leflunomide, # user | 15 (15) | 14 (23) | 0.23 |
| Anti-TNFα, # user | 21 (21) | 10 (16) | 0.43 |


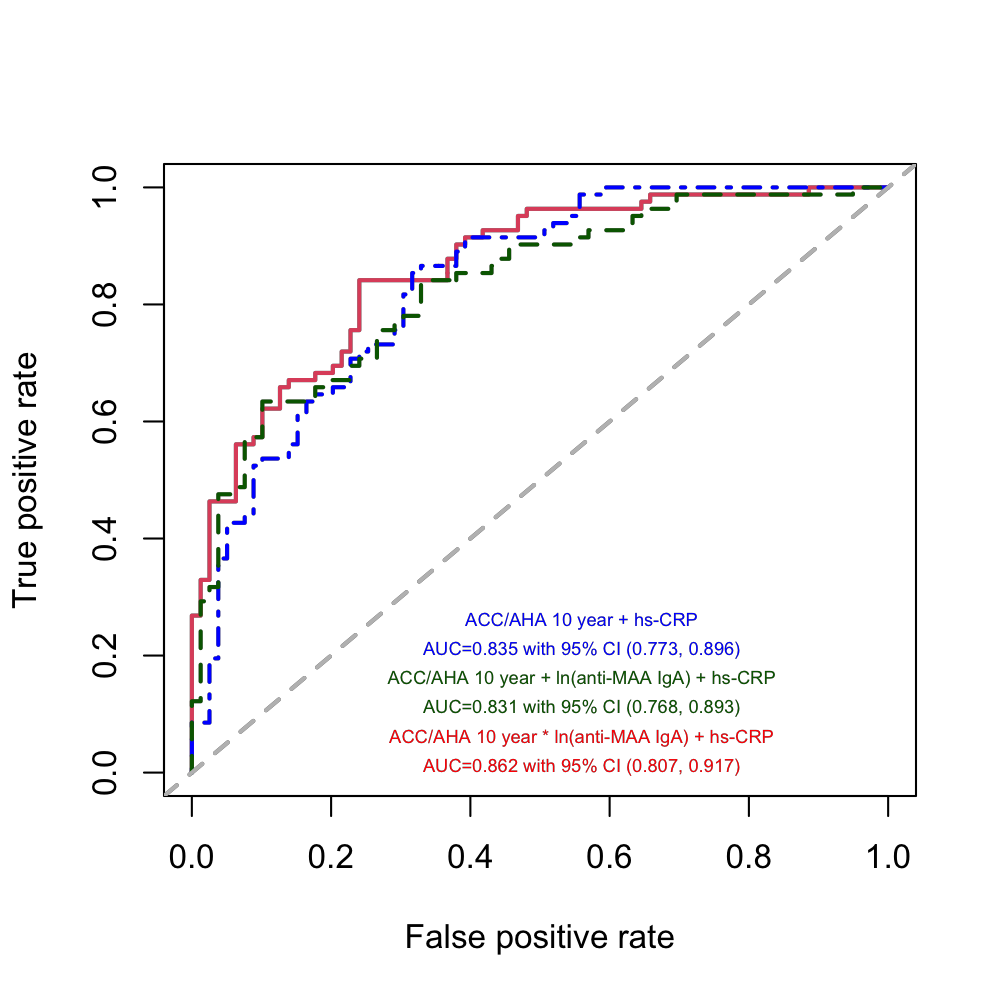


**Supplemental Figure 1.** Receiver operating characteristic curve for prediction of presence of coronary calcium among patients with rheumatoid arthritis. Blue line represents the curve for ACC/AHA 10-year risk score plus high sensitivity CRP (hs-CRP). Green line represents the curve for ACC/AHA 10-year risk score plus natural log-transformed anti-MAA IgA serum concentration plus hs-CRP. Red line represents the curve for the interaction of ACC/AHA 10-year risk score with natural log-transformed anti-MAA IgA serum concentration plus hs-CRP.


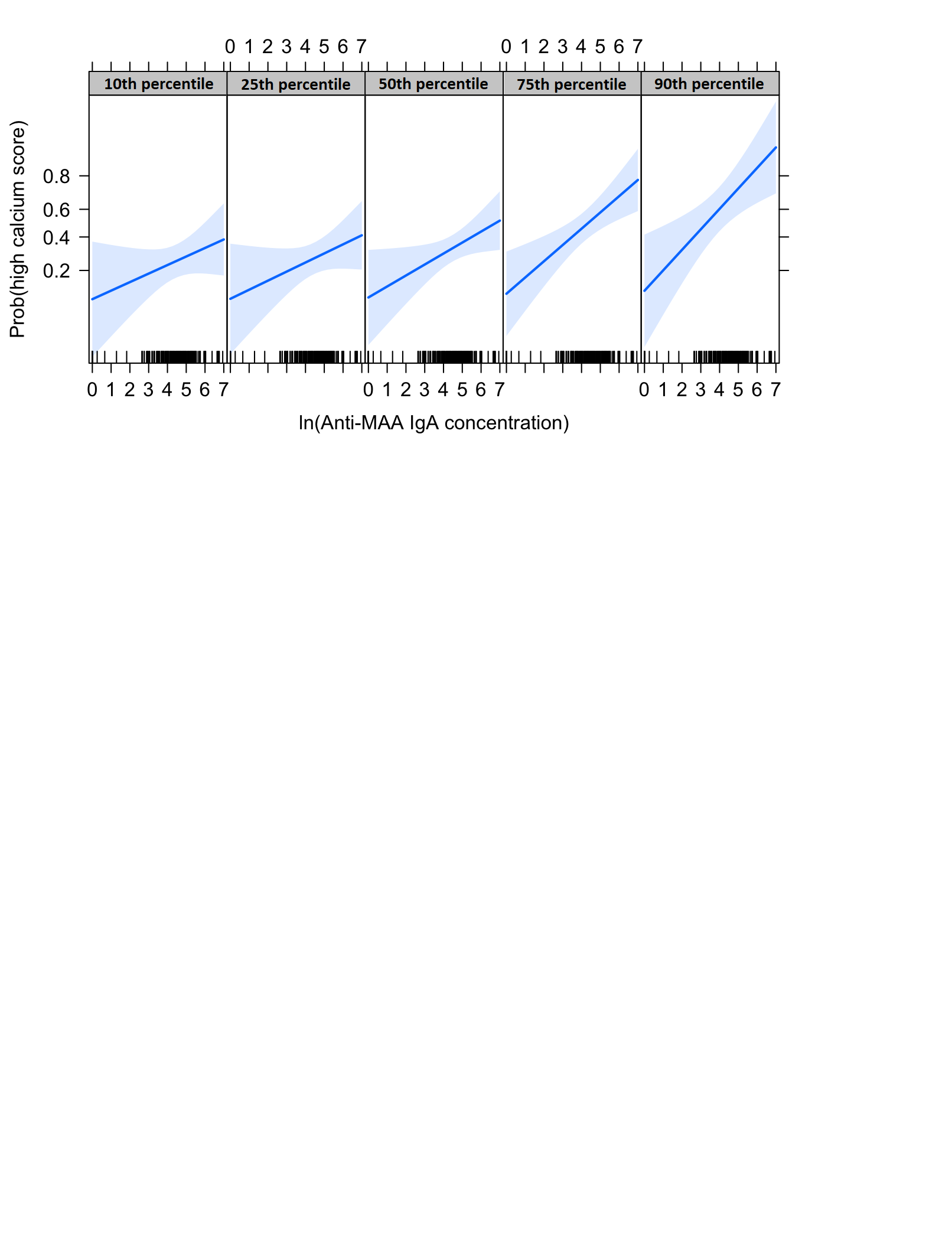


**Supplemental Figure 2.** The interaction between anti-MAA IgA concentration and ACC/AHA 10-year risk score in predicting the presence of high coronary artery calcium. Percentiles refer to the ACC/AHA 10-year risk score percentile of the RA patients. At higher ACC/AHA 10-year risk score, shown as 75^th^ and 90^th^ percentiles, presence of elevated anti-MAA IgA was associated with an amplified probability of high coronary artery calcium.


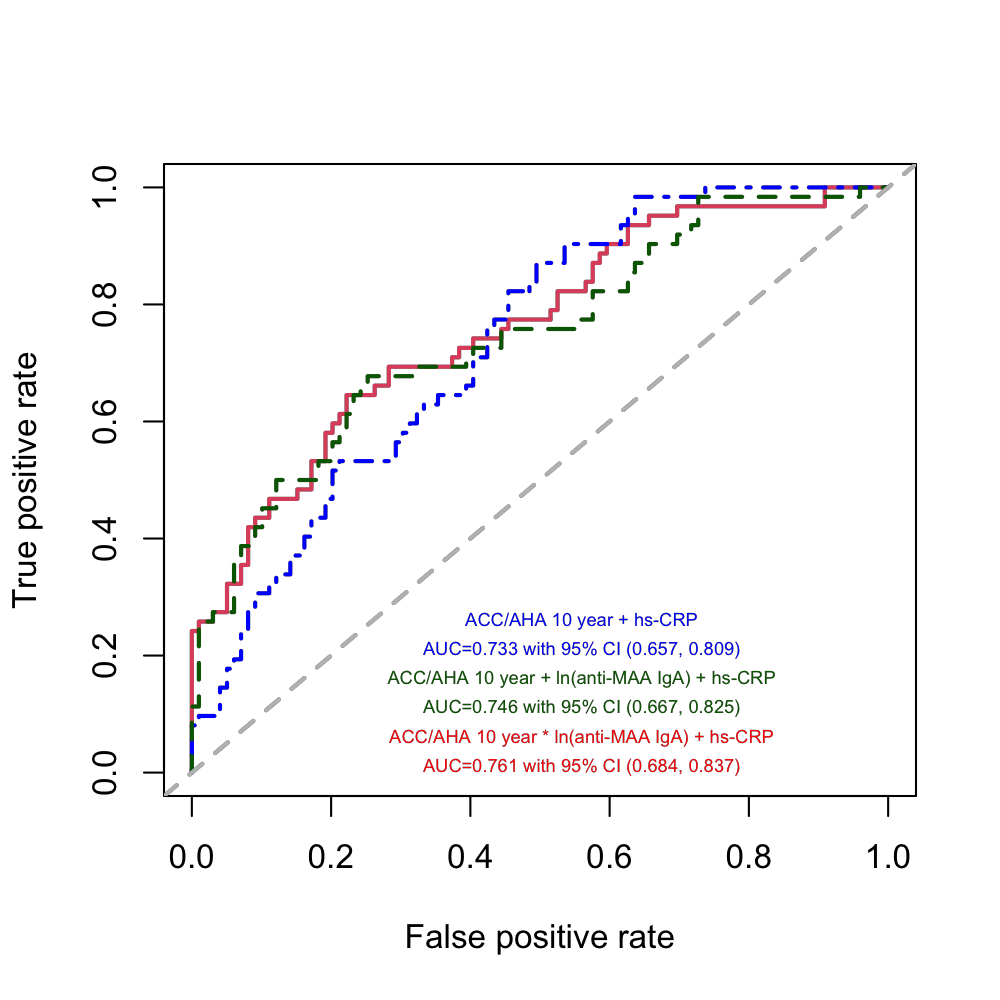


**Supplemental Figure 3.** Receiver operating characteristic curve for prediction of presence of high coronary calcium among patients with rheumatoid arthritis. Blue line represents the curve for ACC/AHA 10-year risk score plus high sensitivity CRP (hs-CRP). Green line represents the curve for ACC/AHA 10-year risk score plus natural log-transformed anti-MAA IgA serum concentration plus hs-CRP. Red line represents the curve for the interaction of ACC/AHA 10-year risk score with natural log-transformed anti-MAA IgA serum concentration plus hs-CRP.
